# Response Assessment and Prediction of Progression-Free Survival by ^68^Ga-PSMA-11 PET/CT Based on Tumor-to-Liver Ratio (TLR) in Patients with mCRPC Undergoing ^177^Lu-PSMA-617 Radioligand Therapy

**DOI:** 10.3390/biom11081099

**Published:** 2021-07-26

**Authors:** Fadi Khreish, Mona Wiessner, Florian Rosar, Zaidoon Ghazal, Amir Sabet, Stephan Maus, Johannes Linxweiler, Mark Bartholomä, Samer Ezziddin

**Affiliations:** 1 Department of Nuclear Medicine, Saarland University, 66421 Homburg, Germany; s8mowies@stud.uni-saarland.de (M.W.); florian.rosar@uks.eu (F.R.); zaidoon.ghazal@uks.eu (Z.G.); stephan.maus@uks.eu (S.M.); mark.bartholomae@uks.eu (M.B.); samer.ezziddin@uks.eu (S.E.); 2 Department of Nuclear Medicine, Frankfurt University, 60590 Frankfurt am Main, Germany; amir.sabet@kgu.de; 3 Department of Urology, Saarland University, 66421 Homburg, Germany; johannes.linxweiler@uks.eu

**Keywords:** metastatic castration-resistant prostate cancer (mCRPC), Lutetium-177, PSMA-617, radioligand therapy, ^68^Ga-PSMA-11 PET/CT, molecular imaging-based response assessment, tumor-to-liver ratio (TLR)

## Abstract

At present, little is known about the molecular imaging-based response assessment of prostate-specific membrane antigen (PSMA)-targeted radioligand therapy with ^177^Lutetium (^177^Lu-PSMA-617 RLT) in metastatic castration-resistant prostate cancer (mCRPC). Our study evaluated the response to RLT using both molecular imaging and biochemical response assessments, and their potential prediction of progression-free survival (PFS). Fifty-one consecutive patients given two cycles of RLT at 6-week intervals were analyzed retrospectively. ^68^Ga-PSMA-11 PET/CT was obtained about 2 weeks prior to the first and 4–6 weeks after the second cycle. Molecular imaging-based response using SUV_peak_ and tumor-to-liver ratio (TLR) was determined by modified PERCIST criteria. ∆TLR and ∆SUV were significantly correlated with ∆PSA (*p <* 0.001, each). After a median follow-up of 49 months, the median PFS (95% CI) was 8.0 (5.9–10.1) months. In univariate analysis, responders showing partial remission (PR_PSA_ and PR_TLR_) had significantly (*p <* 0.001, each) longer PFS (median: 10.5 and 9.3 months) than non-responders showing either stable or progressive disease (median: 4.0 and 3.5 months). Response assessment using SUV_peak_ failed to predict survival. In multivariable analysis, response assessment using TLR was independently associated with PFS (*p* < 0.001), as was good performance status (*p* = 0.002). Molecular imaging-based response assessment with ^68^Ga-PSMA-11 PET/CT using normalization of the total lesion PSMA over healthy liver tissue uptake (TLR) could be an appropriate biomarker to monitor RLT in mCRPC patients and to predict progression-free survival (PFS) of this treatment modality.

## 1. Introduction

Despite advances in early detection and therapy, a significant fraction of patients with prostate cancer develops lethal metastatic castration-resistant prostate carcinoma (mCRPC) [1]. In the last decade, many life-prolonging therapy options, such as chemo-therapy (docetaxel and cabazitaxel, each with survival benefit of 2.4 months compared to mitoxantrone), androgen receptor targeting agents (enzalutamide with survival benefit of 4.8 months and abiraterone with 3.9 months, each compared to placebo), and ^223^Ra therapy (with survival benefit of 2.8 months compared to placebo) have been approved for the treatment of men with mCRPC [2,3,4,5,6]. More recently, studies using ^177^Lu-labeled prostate specific membrane antigen (PSMA) ligands such as PSMA-617, called ^177^Lu-PSMA radioligand therapy (RLT), have shown encouraging results in these patients [7,8,9,10]. The assessment of response to these treatments still relies on biochemical parameters, such as prostate specific antigen (PSA) and conventional imaging modalities, including computed tomography (CT), magnetic resonance tomography (MRI), and bone scintigraphy [11]. However, these conventional imaging tools have limited diagnostic value in the advanced stage of disease, especially in the response evaluation of bone metastases, which could show more sclerotic changes, even after successful treatment and therapy response. Thus, there is significant demand for a reliable imaging methodology to accurately monitor treatment, especially for ^177^Lu-PSMA-617 RLT in the setting of mCRPC. The response assessment by positron emission tomography/computed tomography (PET/CT) using ^18^F-fluorocholine has been investigated in patients with mCRPC treated with docetaxel [12], abiraterone [13], or enzalutamide [14], and showed the ability to predict clinical outcome beyond the PSA response. In recent years, ^68^Ga-PSMA-11 PET/CT has gained increasing importance in the management of prostate cancer for initial staging [15], biochemical recurrence [16], detection of metastases in non-metastatic CRPC [17], and screening for ^177^Lu-PSMA-617 RLT. Recently, Violet et al. found a significant correlation between tumoral uptake on pretherapeutic ^68^Ga-PSMA PET and absorbed dose by the tumor, estimated on a ^177^Lu-PSMA-617 whole-body scan using an automated voxelized dosimetry tool [18]. However, little is known about the impact of ^68^Ga-PSMA-11 PET/CT on response assessment of different therapy options in mCRPC, especially for ^177^Lu-PSMA-617 RLT [19,20].

The aim of this study was to evaluate ^68^Ga-PSMA-11 PET-derived parameters (SU-Vpeak and the normalization over the healthy liver tissue uptake/tumor-to-liver ratio/TLR) as potential tools for monitoring ^177^Lu-PSMA-617 RLT. Molecular imaging-based response assessment using the modified PERCIST criteria was compared with biochemical response assessment using the established biomarker serum PSA. Furthermore, the potential value of these PET-derived parameters for the prediction of progression-free survival outcome was evaluated.

## 2. Materials and Methods

### 2.1. Patients and Ethics

The study population included patients with late-stage/end-stage mCRPC, who underwent ^177^Lu-PSMA-617 RLT at our institution. Inclusion requirements for this study were histologically confirmed mCRPC, at least 2 cycles of ^177^Lu-PSMA-617 RLT, ^68^Ga-PSMA-11 PET/CT about 2 weeks before the first and 4–6 weeks after the second cycle of ^177^Lu-PSMA-617 RLT, and availability of clinical outcome data. Furthermore, intense PSMA expression of all tumor lesions on pre-treatment ^68^Ga-PSMA-11 PET/CT, defined visually, as tumoral uptake higher than liver uptake was required. The standard decision approach in choosing ^177^Lu-PSMA-617 RLT was based on multidisciplinary tumor board discussion and was in accordance with German healthcare reimbursement specifications.

This therapy was performed on a compassionate use basis under the German Pharmaceutical Act §13 (2b). Patients gave written consent after being thoroughly informed about the risks and potential side effects of this intervention. Additionally, patients consented to publication of any resulting data in accordance with the Declaration of Helsinki. The analysis was approved by the local institutional review board (ethics committee permission number 140/17).

### 2.2. ^177^Lu-PSMA-617 RLT

Each patient received two cycles of ^177^Lu-PSMA-617 RLT with mean cumulative administered activities of 12.9 GBq (range: 9.1–16.9). Patients continued their androgen deprivation therapy (ADT). Patients were excluded if they underwent changes in ADT regimens between scans at baseline and restaging after two cycles. ADT, especially enzalutamide, can alter the PSMA expression in prostate carcinoma cells [21] and may therefore falsify SUV measurements.

PSMA-617 was obtained from ABX advanced biochemical compounds GmbH (Radeberg, Germany) and ^177^Lu from IDB Holland BV (Baarle-Nassau, The Netherlands). Production and quality control of ^177^Lu-PSMA-617 was accomplished analogously to a published methodology [9]. For a typical labeling, 150 μg (143 nmol) PSMA-617 was used for 6 GBq of ^177^Lu. Radiochemical yields and purity of the radiotracer were ≥99%.

### 2.3. ^68^Ga-PSMA-11 PET-CT Imaging and Data Acquisition

A mean activity of ^68^Ga-PSMA-11 of 120 ± 19 MBq was administered intravenously, followed by a 500 mL infusion of NaCl 0.9%. No diuretics were applied. The mean time between injection and PET acquisition was approximately 60 min according to standard procedures for prostate cancer imaging [22]. PET/CT datasets were acquired on a Biograph 40 mCT PET/CT scanner (Siemens Medical Solutions, Knoxville, TN, USA) (acquisition time: 3 min/bed position; extended FOV: 21.4 cm (TrueV), slice thickness: 3.0 mm) with EANM Research Ltd. accreditation. A standard LD spiral CT was acquired for attenuation correction and anatomical localization using an X-ray tube voltage of 120 keV and a modulation of the tube current applying CARE Dose4D with a reference tube current of 50 mAs. CT images were reconstructed as a 512 × 512 matrix with an increment of 3 mm and a slice thickness of 5.0 mm. The PET images were iteratively reconstructed using the three-dimensional OSEM (ordered-subset expectation maximization) algorithm with 3 iterations, 24 subsets, and with Gaussian filtering to a transaxial resolution of 5 mm at full-width at half-maximum (FWHM). Attenuation correction was performed using the low-dose non-enhanced CT data.

### 2.4. Biochemical Response Assessment

Biochemical response to ^177^Lu-PSMA-617 RLT was measured in terms of serum PSA level. Biochemical partial response (PR_PSA_) was defined as a decrease in serum PSA level of ≥50% from the baseline value. Stable disease (SD_PSA_) was defined as an intermediate change of serum PSA level between −50% and +25%. Progressive disease (PD_PSA_) was defined using the Prostate Cancer Working Group 3 criteria (PCWG3) [11] as an increase in serum PSA level of ≥25% and at least 2 ng/mL.

### 2.5. Molecular Imaging-Based Response Assessment

The response to ^177^Lu-PSMA-617 RLT was assessed by ^68^Ga-PSMA-11 PET/CT. Assuming that ^177^Lu-PSMA-617 RLT had different effects on metastases in different organs, as reported by Kulkarni et al. [23] with lymph node metastases responding better to this treatment than bone metastases, we included a five-organ system per patient in the response assessment, as previously described by Seitz et al. [24]. These comprised lymph nodes, bones, liver, prostate or prostate bed, and other organs. For each organ, the three lesions with the highest PSMA uptake on ^68^Ga-PSMA-11 PET/CT were identified as target lesions and included in this analysis. Using the volume region of interest, SUV_peak_ of the tumor lesions and SUV_mean_ of healthy liver tissue were measured. Then, the sum of the SUV_peak_ of all included lesions was divided by the SUV_mean_ of the liver to calculate the tumor-to-liver ratio (TLR). This method was applied to each individual patient at each time point (before the first and after the second cycle of RLT).

For molecular imaging-based response assessment, the PET Response Criteria in Solid Tumors (PERCIST) 1.0 were slightly modified [25] and then used to interpret post treatment changes. Here, the following definitions were made: Molecular imaging-based partial response (PR_SUV_ or PR_TLR_) represents a decrease of >30% of summed SUV_peak_ or TLR, respectively. Progressive disease (PD_SUV_ or PD_TLR_) was defined as an increase of >30% of SUV_peak_ or TLR, respectively, or appearance of any new lesion. A change within the range of +30% and −30% was considered a stable disease (SD_SUV_ or SD_TLR_).

### 2.6. Statistics and Survival Assessment

Data on patient characteristics, treatment-related data, and response analyses are presented descriptively and analyzed regarding association with progression-free survival (PFS). PFS was defined as the interval from the start of ^177^Lu-PSMA-617 RLT to documented PSA progression (as defined using the Prostate Cancer Working Group 3 criteria (PCWG3) or death (cut-off date: 30 March 2021). Patients were independently categorized as responder (partial response) or non-responder (stable or progressive disease), either by molecular imaging-based response or biochemical response assessment.

The examined patient, disease, and treatment characteristics included age, performance status, hemoglobin, alkaline phosphatase (ALP), prior chemotherapy, presence of visceral metastasis, diffuse bone marrow involvement, tumor burden on ^68^Ga-PSMA-11 PET/CT (low-moderate vs. high) at baseline, and PSA levels. Total tumor burden at baseline were visually classified in low, moderate, or high in analogy to Gaertner et al. [26]. Characteristics furthermore included the cumulative ^177^Lu-PSMA-617 activity of the first 2 cycles of RLT, PSA response after two cycles, and the molecular imaging-based response by modified PERCIST criteria starting with the first ^177^Lu-PSMA-617 administration. Variables were analyzed categorically. For age, performance status, hemoglobin level, ALP level, and PSA level at the start of ^177^Lu-PSMA-617 RLT, respective arbitrary cut-offs of 65 years, ECOG 1, 12 g/dL, 220 U/L, and 230 ng/mL were employed. Because of inhomogeneously administered ^177^Lu-PSMA-617 activities, we divided the study cohort into two groups: patients who received more than 13 GBq, and those that received equal to or less than 13 GBq cumulative activities in the first 2 cycles of RLT.

PFS was analyzed using Kaplan–Meier statistics. To identify predictors of this endpoint, multivariable Cox proportional-hazards modeling was performed using a stepwise model by backward elimination. Factors with moderate significance (*p <* 0.15) in univariate analysis were included in the multivariable analysis. Correlation between molecular imaging-based response (∆TLR, ∆SUV) and biochemical response (∆PSA) was calculated using Spearman’s rank correlation test. Cohen’s κ was used to assess the degree of agreement among the response variables. The significance level for all tests was *p* < 0.05. SPSS version 23 (SPSS Inc., Chicago, IL, USA) and Prism 8 software (GraphPad Software, San Diego, CA, USA) were used.

## 3. Results

The data of 51 patients who met the inclusion criteria were retrospectively analyzed (characteristics summarized in Table 1). A total of 322 tumor lesions on screening ^68^Ga-PSMA-PET/CT in 51 patients were identified as target lesions in the defined five-organ system and evaluated in this study. These included 142 bone metastases (in 48 patients), 110 lymph node metastases (in 40 patients), 30 liver metastases (in 12 patients), 22 lesions in the prostate bed (in 22 patients), and 18 other metastases (in 10 patients).

**Figure 1:**
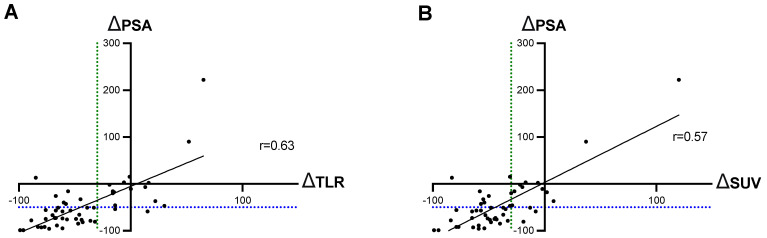
Correlation between ∆PSA and (**A**) ∆TLR and (**B**) ∆SUV. The blue dotted line is localized at −50% PSA change and the green dotted line at −30% of TLR/SUV.

The Spearman test showed a significant correlation between changes in ∆PSA and ∆SUV, and between ∆PSA and ∆TLR, in the comparison of the parameters before and after two cycles of RLT (*p <* 0.001, each). The correlation was slightly better between ∆PSA and ∆TLR than the correlation between ∆PSA and ∆SUV (rTLR = 0.63 vs. rSUV = 0.57), Figure 1.

### 3.1. Biochemical and Molecular-Imaging Response Assessment

**Figure 2:**
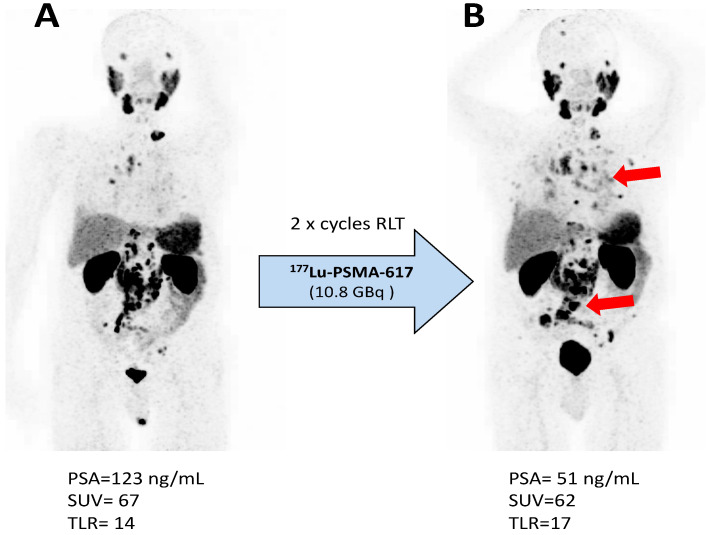
Progression with appearance of new metastases illustrated by ^68^Ga-PSMA-11 PET/CT maximum-intensity projection images (**A**) before and (**B**) 5 weeks after the second cycle of ^177^Lu-PSMA-617 RLT in a man with mCRPC. According to biochemical response based on serum PSA levels, the patient showed a partial remission.

**Figure 3:**
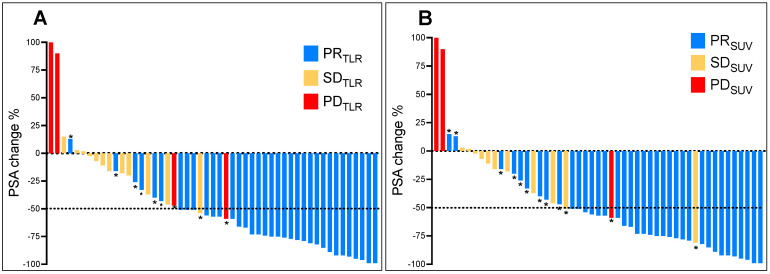
Waterfall plots illustrating the relative changes in standard biochemical parameters (PSA) after two cycles of ^177^Lu-PSMA-617 RLT compared to the assessment in molecular-imaging based response with TLR (**A**) and SUV (**B**). Values marked with * highlight the discordant cases.

**Figure 4:**
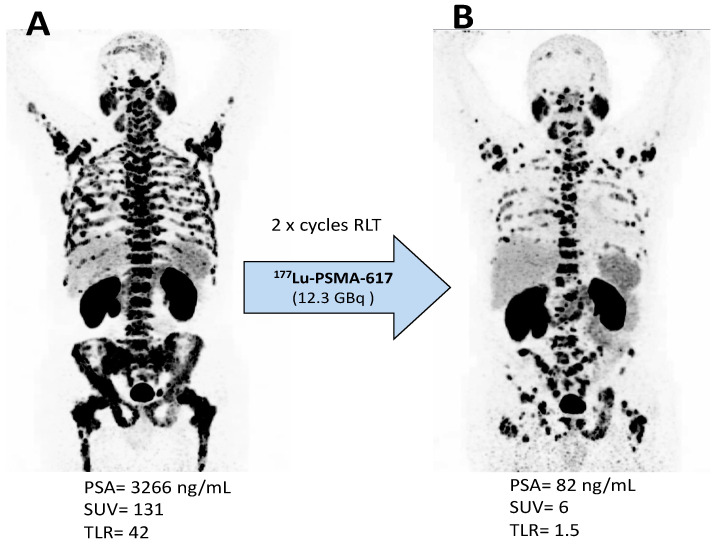
Concordant biochemical and molecular imaging-based response as illustrated by ^68^Ga-PSMA-11 PET/CT maximum-intensity projection images (**A**) before and (**B**) 6 weeks after the second cycle of ^177^Lu-PSMA-617 RLT in a man with mCRPC. The patient was classified as having partial remission by each of PSA, SUV, and TLR.

Thirty-one patients (61%) showed partial biochemical response (PR_PSA_), 18 patients (35%) stable disease (SD_PSA_), and two patients (4%) progressive disease (PD_PSA_). Using molecular-imaging based response assessed by SUV_peak_, 37 patients (73%) showed partial response (PR_SUV_), 12 patients (23%) stable disease (SD_SUV_), and two patients (4%) progressive disease (PD_SUV_). Response assessment using TLR gave similar results, with PR_TLR_ in 35 patients (69%), SD_TLR_ in 13 patients (25%), and PD_TLR_ in three patients (6%). A comparison of the used response assessment methods showed a moderate agreement (Cohen’s κ = 0.45) with a concordance of 76% (39/51) between SUV and PSA, and a substantial agreement (Cohen’s κ = 0.61) with a concordance of 82% (42/51) between TLR and PSA. The concordance between TLR and SUV was 88% (45/51). With the exception of two patients, all subjects showing partial remission by PSA also showed partial remission by TLR. One of these two patients was classified by molecular imaging-based response as having disease progression due to appearance of new metastases (Figure 2) and the other patient as stable disease. The majority of discrepancies were found in patients with stable disease by PSA (Figure 3A). Two patients showed concordance with progressive disease by both methods. With the exception of three patients, all who showed partial remission by PSA also showed partial remission by SUV. Of these, one patient was classified as having disease progression due to the appearance of new metastases (Figure 2) and two patients as having stable disease. The majority of discrepancies were found in patients with stable disease by PSA (Figure 3B). Two patients showed concordance with progressive disease by both methods. Representative structural responses to ^177^Lu-PSMA-617 RLT are shown in Figure 2 and Figure 4.

### 3.2. Survival Analysis

Median (minimum–maximum) follow-up was 49.0 (6.0–64.4) months following the first ^177^Lu-PSMA-617 RLT administration. Forty patients (78.4%) died by the end of the study. All deaths were mCRPC related. No treatment-related mortality was observed. The median (95% CI) OS was 14.7 (11.2–18.2) months. Disease progression events were documented in 44 men (86.3%). The median (95% CI) PFS was 8.0 (5.9–10.1) months. Two factors related to response to ^177^Lu-PSMA-617 RLT and one factor related to patient’s characteristics were significantly associated with PFS in univariate analysis (Table 2).

**Figure 5:**
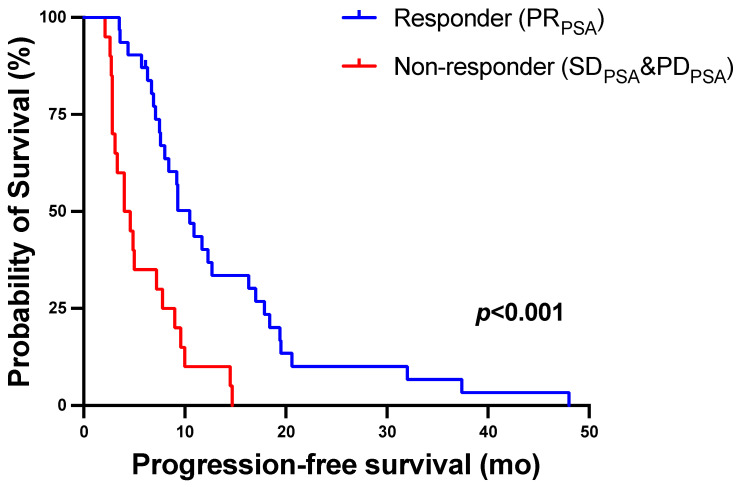
Kaplan–Meier curves for PFS stratified by biochemical response using serum PSA change after two cycles of ^177^Lu-PSMA-617 RLT.

Responders showing biochemical partial remission (PR_PSA_) after two cycles of ^177^Lu-PSMA-617 RLT had significantly longer PFS than non-responders (SD _PSA_ or PD _PSA_) with a median PFS (95% CI) of 10.5 (8.2–12.8) versus 4.0 (2.1–5.9) months (*p* < 0.001, log-rank test). The corresponding Kaplan–Meier curves are shown in Figure 5.

**Figure 6:**
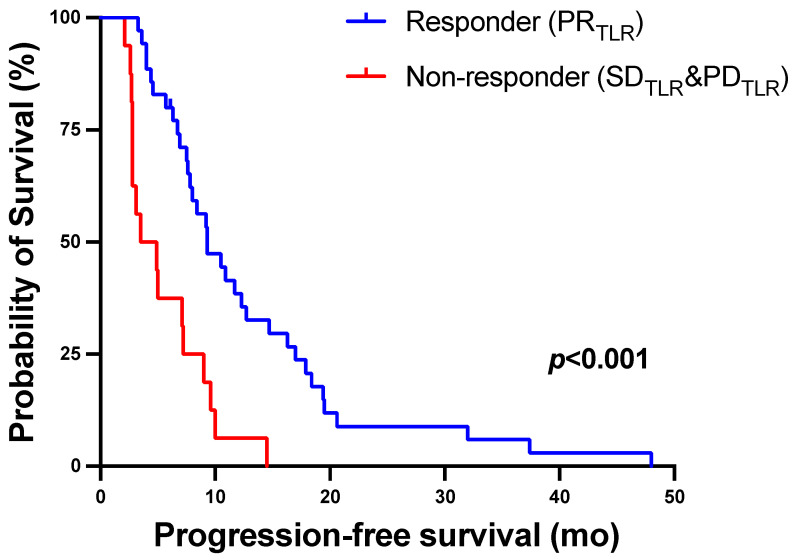
Kaplan–Meier curves for PFS stratified by molecular-imaging response assessment using TLR after two cycles of ^177^Lu-PSMA-617 RLT.

Response on molecular imaging using TLR after two cycles of ^177^Lu-PSMA-617 RLT was associated with longer PFS (Table 2). Patients with PR_TLR_ had a median PFS (95% CI) of 9.3 (6.9–11.7) months versus 3.5 (0.9–6.1) months for non-responders who showed SD_TLR_ or PD_TLR_ (*p* ≤ 0.001, log-rank test). The corresponding Kaplan–Meier curves are shown in Figure 6.

**Figure 7:**
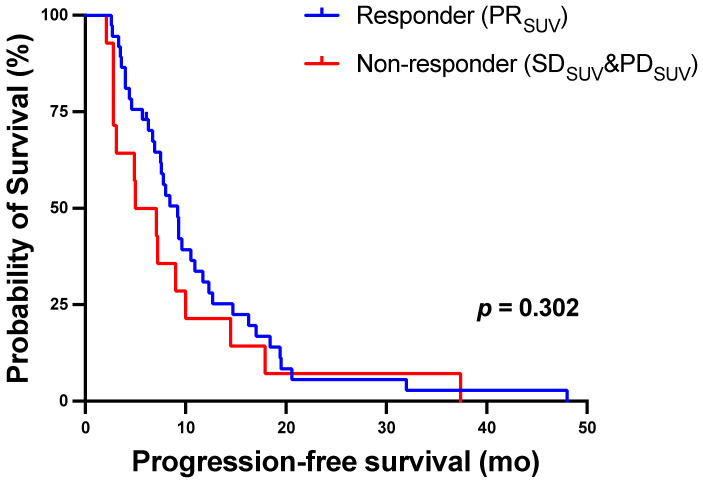
Kaplan–Meier curves for PFS stratified by molecular-imaging response assessment using SUV_peak_ after two cycles of ^177^Lu-PSMA-617 RLT.

There was no significant outcome difference between responders and non-responders in the molecular-imaging based response assessment using SUV_peak_ with a median PFS of 9.2 (7.5–10.9) months versus 5.0 (0.9–9.0), for responders and non-responders, respectively, *p* = 0.302. The corresponding Kaplan–Meier curves are shown in Figure 7. The general condition of the patient at baseline was also related to PFS. Median (95% CI) PFS was 9.3 (7.4–11.2) months in men with ECOG 0-1 versus 4.4 (2.4–6.5) months in those with ECOG > 1 (*p* ≤ 0.001, log-rank test).

In the multivariable analysis, partial response in the molecular-imaging based response assessment using TLR (PR_TLR_) remained an independent predictor for PFS with a HR (95% CI) of 0.27 (0.14–0.54; *p <* 0.001). Preserved patient performance score (ECOG 0-1) also remained as an independent predictor of PFS with a HR (95% CI) of 0.26 (0.12–0.56; *p* = 0.002). Biochemical response based on serum PSA levels did not remain independently significant in multivariable analysis (*p* = 0.091).

## 4. Discussion

The aim of this study was to investigate the feasibility of ^68^Ga-PSMA-11 PET/CT for response assessment and outcome prediction of ^177^Lu-PSMA-617 RLT given to patients with mCRPC. For this purpose, molecular imaging-based response and biochemical response after two cycles of ^177^Lu-PSMA-617 RLT in 51 patients were evaluated. Molecular imaging-based response using ^68^Ga-PSMA-11 PET-derived parameters (SUV_peak_ and tumor-to-liver ratio: TLR) was assessed by a five-organ system and modified PERCIST criteria. Biochemical response was measured in terms of serum PSA level and assessed using PCWG3.

This is the first study to demonstrate that response assessment based on molecular imaging with ^68^Ga-PSMA-11 PET/CT using normalization of tumoral PSMA uptake over healthy liver tissue (TLR) is a predictor of outcome (PFS) in patients with late-stage mCRPC treated with ^177^Lu-PSMA-617 RLT. After two cycles of ^177^Lu-PSMA-617 RLT, responders (PR_TLR_) had a significantly lower risk for disease progression or death in comparison to non-responders (SD_TLR_ or PD_TLR_). On the contrary, molecular imaging response assessment using SUV_peak_ failed to predict PFS in univariate analysis; responders (PR_SUV_) had a median PFS of 9.2 months versus 5.0 months in non-responders (SD_SUV_ or PD_SUV_), *p* = 0.302. Recently published studies that also addressed the role of ^68^Ga-PSMA-11 PET/CT in assessing response to ^177^Lu-PSMA-617 RLT similarly reported no predictive value of SUV alone, either for response or for survival [19,20]. One potential explanation is the known existence of error sources for SUV measurements, such as incubation time variations, inaccurate weight assessments, and extravasation of injected activity, resulting in suboptimal test/retest accuracy [27]. These shortcomings were mostly overcome by normalization of SUV measurements over the uptake of a reference organ. In our study, healthy liver tissue was chosen as the reference region because of little potential ^177^Lu-PSMA-617 RLT related toxicity and possible changes in tracer uptake of baseline versus interim PET compared to other sites of physiologic uptake, such as kidneys or salivary glands, as known from PSMA dosimetry reports [28]. Moreover, liver SUV assessments are facilitated by the generally homogenous physiological PSMA expression and the ease of obtaining a representative area for VOI placement, even in patients with liver metastases. The chosen method of SUV normalization over liver proved to have a high impact regarding survival prediction, which might be seen to be analogous to the TLR parameter in FDG PET for lymphoma response assessment, which is well established and superior to merely (non-normalized) SUV-based response classifications [29].

Regarding the two molecular imaging biomarkers, SUV_peak_ and newly introduced TLR, the correlation with the biochemical biomarker PSA showed minor differences. In our cohort, we documented a PSA decline >50% after two 2 cycles of ^177^Lu-PSMA-617 RLT in 61% of patients. By molecular imaging-based response assessment, partial response was observed in 73% of patients using SUV_peak_ and in 68% of patients using TLR. These results are in agreement with previously published reports showing efficacy of this treatment modality in advanced mCRPC [8,30,31]. The correlation between ∆SUV_peak_ and ∆PSA was significant, although with only a moderate r value (r_SUV_ = 0.57, *p <* 0.001), and ∆TLR showed slightly a superior correlation with ∆PSA (r_TLR_ = 0.63, *p* ≤ 0.001). The resulting response categories (PR, SD, PR) were again associated with slightly superior concordance rates for TLR over SUV_peak_: 76% (SUV_peak_ vs. PSA) and 82% (TLR vs. PSA). Similar concordance values of 65–87% between PSA and SUV changes have been reported in other studies with different cohorts of patients with prostate cancer [31,32]. However, to our knowledge, this is the first study to show the correlation between PSA and TLR.

Serum PSA level is the most widely used parameter for treatment monitoring in daily routine and in prospective trials dealing with advanced prostate cancer. In addition, in ^177^Lu-PSMA RLT studies, ∆PSA during or at the end of therapy is a commonly used biomarker to evaluate treatment success. In a phase II prospective trial of ^177^Lu-PSMA-617 in mCRPC, Violet et al. reported that patients achieving a PSA decline >50% had a longer PFS than those without this decline [33]. Our data confirms this predictive power of PSA decline in the early response assessment. However, in multivariable analysis, this parameter lost the independent predictive impact, apparently due to its strong association with ∆TLR, which proved slightly superior to ∆PSA for prediction of PFS; that is, response by PSA and response by TLR were very similar in our cohort and thus not independent from each other. The more accurate predictor of PFS, ∆TLR, remained as an independent predictor, whereas ∆PSA was removed as an “independent predictor” within multivariable analysis. Nevertheless, ∆PSA was significantly associated with PFS in univariate analysis (*p <* 0.001). One should note that serial PSA measurements may sometimes miss disease progression, as concluded by authors from a large study (post hoc analysis of the PREVAIL trial) including *n* = 872 mCRPC patients undergoing enzalutamide treatment versus placebo [34]. In their conclusion, they state that “Non-rising PSA at radiographic progression is a common phenomenon in mCRPC patients treated with enzalutamide”. Furthermore, “Therefore, a disease monitoring strategy that includes imaging not entirely reliant on serial serum PSA measurement may more accurately identify disease progression”. Recently Michalski et al. published data regarding assessment of the response to ^177^Lu-PSMA radioligand therapy using modified PSMA PET Progression Criteria [35]. They found that progression according to modified PPP criteria was a significant prognostic marker for OS (*p* < 0.001) with a hazard ratio (HR) of 15.5 (95% CI 3.4–70.2). In contrast, the response of serum PSA level was not significant (*p* = 0.12). However, there are some methodological differences with our study; namely, they analyzed the response using serum PSA at the end of therapy (in our study, after two cycles) and stratified the patients as progressive versus non-progressive (in our study, as responders versus non-responders), and analyzed the predictor for OS (in our study, for PFS). Although not shown in this study, whether a less-marked PSA decline (less than 50%) as a cut-off for response may better distinguish associated PFS may eventually be shown by investigations with larger patient cohorts.

In addition, the patients’ performance status was an independent predictor of PFS in our study. Patients with a good general condition, defined as ECOG 0-1 at the start of ^177^Lu-PSMA-617 RLT, had significantly longer PFS than those with a worsened performance score (ECOG > 1). These results are in accordance with the literature [36] and with our previous study of hepatic metastasized mCRPC patients treated with ^177^Lu-PSMA-617 RLT [37].

## 5. Limitation

The main limitations of this study are its retrospective design and the small number of patients. Because interim staging after two cycles of ^177^Lu-PSMA-617 RLT was performed with PET-CT consisting of non-contrast enhanced low-dose CT, it was not possible to define the response using Response Criteria in Solid Tumors. Furthermore, the five-organ system includes metastases from different sites but does not reflect the total viable tumor burden. Therefore, using total tumor volume parameters, especially in patients with mixed responses, might be of advantage by reflecting the total viable PSMA expressing tumor burden in contrast to selected target lesions. Another limitation that may affect liver SUV measurements is the presence of disseminated liver metastasis, but this is rare in the mCRPC setting.

## 6. Conclusions

^68^Ga-PSMA-11 PET-derived response assessment based on normalization of tumoral PSMA expression to normal liver tissue (TLR) may be an appropriate biomarker for monitoring ^177^Lu-PSMA-617 RLT in mCRPC patients and predicting survival outcome (PFS) of this treatment modality. Our study indicates the superiority of using liver-normalized tumor SUV values for molecular response assessment in this setting compared to the use of standard tumor SUV measurements, which are widely practiced at present. However, prospective studies with larger patient cohorts are needed to confirm these initial findings.

## Figures and Tables

**Table 1:** Patient characteristics.

Patient Characteristics	Value, No. (%)
Number of Patients	51
**Number of therapeutic cycles**	2
**Median Activity (Range) (GBq)**	12.9 (9.1–16.9)
**Age**	
Median (Range)	74.5 (49–89)
**ECOG**	
0–1	40 (78%)
≥1	11 (22%)
**Baseline PSA in ng/mL**	
Median (Range)	235.5 (0.21–3266)
**Baseline AP (U/L)**	
Median (Range)	200 (133–1753)
AP ≥ 220	10 (20%)
**Previous therapies**	
Surgery (Prostatectomy)	23 (45%)
ADT	51 (100%)
External beam radiotherapy of prostate or prostate bed	26 (51%)
Docetaxel or Cabazitaxel	36 (71%)
≥2 lines of taxanes	15 (29%)
Abiraterone or Enzalutamide	47 (92%)
Both (Enzalutamide and Abiraterone)	22 (43%)
^223^Ra therapy	10 (20%)
**Patterns of metastatic spread**	
Predominant lymph nodes	12 (24%)
Predominant bone	43 (84%)
Visceral organs	18 (35%)

**Table 2:** Relationship of PFS with selected factors in patients with mCRPC treated with ^177^Lu-PSMA-617.

Variable Category or Categories	*n* (%)	PFS, Months	Univariate Analysis		Multivariable Analysis	
		(95% CI)	HR (95% CI)	*p*	HR (95% CI)	*p*
**Total**	51 (100%)	8.0 (5.9–10.1)				
**Age**						
>65 yr	42 (83%)	8.4 (6.4–10.4)	-	0.724	-	-
≤65 yr	9 (17%)	5.7 (3.7–7.7)	-			
**Performance status**						
ECOG ≤ 1	40 (78%)	9.3 (7.4–11.2)	0.25 (0.12–0.54)	**<0.001**	0.26 (0.12–0.56)	**0.002**
ECOG > 1	11 (22%)	4.4 (2.4–6.5)				
**Hemoglobin**						
HB > 13 g/dL	21 (41%)	8.0 (3.7–12.3)	-	0.782	-	-
Hb ≤ 12 g/dL	30 (59%)	7.8 (5.5–10.1)				
**ALP**						
<220 U/L	39 (76.5%)	12.3 (9.7–14.9)		0.249		
≥220 U/L	12 (23.5%)	9.2 (4.6–13.8)				
**Prior chemotherapy**						
No	15 (29%)	9.3 (7.3–11.3)	-	0.862	-	-
Yes	36 (71%)	7.2 (6.2–8.2)				
**Visceral metastases at basline**						
No	33 (65%)	8.4 (6.2–10.6)	-	0.529	-	-
Yes	18 (35%)	6.7 (3.8–9.6)				
**Tumor burden**						
Low-moderate	31 (61%)	9.6 (6.7–12.5)		0.223		
high	20 (39%)	6.7 (2.3–11.1)				
**Diffuse bone marrow involvment at basline**						
Yes	15 (29%)	8.4 (5.2–11.6)	-	0.838	-	-
No	36 (71%)	7.8 (5.7–9.9)				
**PSA level at start of ^177^Lu-PSMA-617 RLT**						
≤230 ng/mL	26 (51%)	8.4 (5.5–11.3)	-	0.757	-	-
>230 ng/mL	25 (49%)	7.6 (6.1–9.1)				
**Cumulative 177Lu-PSMA-617 activity of the first 2 cycles RLT**						
≤13 GBq	27 (53%)	9.6 (6.0–13.2)	-	0.336	-	-
>13 GBq	24 (47%)	7.2 (5.4–9.0)				
**Biochemical response after the first two cycles RLT**						
PR_PSA_	31 (61%)	10.5 (8.2–12.8)	0.29 (0.16–0.56)	**<0.001**	0.24 (0.12–0.49)	0.091
SD_PSA_ or PD_PSA_	20 (39%)	4.0 (2.1–5.9)				
**Molecular-imaging response SUV after the first two cycles RLT**						
PR_SUV_	37 (73%)	9.2 (7.5–10.9)	-	0.302	-	-
SD_SUV_ or PD_SUV_	14 (27%)	5.0 (0.9–9.0)				
**Molecular-imaging response TLR after the first two cycles RLT**						
PR_TLR_	35 (69%)	9.3 (6.9–11.7)	0.27 (0.15–0.53)	**<0.001**	0.27 (0.14–0.54)	**<0.001**
SD_TLR_ or PD_TLR_	16 (31%)	3.5 (0.9–6.1)				

## Data Availability

The datasets used and analyzed during the current study are available from the corresponding author on reasonable request.
